# Neurofilament Light Chain Is a Novel Biomarker for Major Depression and Related Executive Dysfunction

**DOI:** 10.1093/ijnp/pyab068

**Published:** 2021-10-12

**Authors:** Mu-Hong Chen, Yu-Li Liu, Hsiang-Wei Kuo, Shih-Jen Tsai, Ju-Wei Hsu, Kai-Lin Huang, Pei-Chi Tu, Ya-Mei Bai

**Affiliations:** 1 Department of Psychiatry, Taipei Veterans General Hospital, Taipei, Taiwan; 2 Department of Psychiatry, College of Medicine, National Yang Ming Chiao Tung University, Taipei, Taiwan; 3 Department of Medical Research, Taipei Veterans General Hospital, Taipei, Taiwan; 4 Center for Neuropsychiatric Research, National Health Research Institutes, Zhunan, Miaoli County, Taiwan

**Keywords:** Cognitive function, major depressive disorder, neurofilament light chain, proinflammatory cytokines

## Abstract

**Background:**

Evidence suggests that major depressive disorder is related to neuroaxonal injury and that neurofilament light chain (NfL) is a biomarker of neuroaxonal injury. In addition, proinflammatory cytokines have been reported to be associated with major depression and neuroaxonal injury.

**Methods:**

Forty patients with major depression and 40 age- and sex-matched healthy control participants were enrolled for the measurement of NfL and proinflammatory cytokines and assessment of executive function. General linear models were used to examine the association between NfL levels, proinflammatory cytokine levels, and executive function.

**Results:**

Patients with major depressive disorder exhibited significantly higher NfL levels (*P* = .007) than the control participants. NfL levels were positively related to log-transformed levels of tumor necrosis factor-α (*P* = .004). Higher levels of NfL (*P* = .002) and tumor necrosis factor-α (*P* = .013) were associated with greater deficits in executive function.

**Discussion:**

NfL was a novel biomarker for major depressive disorder and related executive dysfunction. Further studies are necessary to elucidate the role of NfL in the pathophysiology of major depression and related cognitive impairment.

**Significance Statement**

Neurofilament light chain (NfL) works as a biomarker of neuroaxonal injury. We analyzed the NfL levels between 40 patients with major depression and 40 age- and sex-matched healthy controls and found NfL levels were significantly elevated in patients with major depressive disorder. We further observed a positive relationship between NfL and tumor necrosis factor (TNF)-α levels. In addition, higher levels of NfL and TNF-α were associated with greater deficits in executive function. Our findings may indicate the important roles of neuroaxonal injury and proinflammatory cytokines in the pathophysiology of major depressive disorder.

Major depressive disorder has been increasingly recognized as a chronic and deteriorating mental illness over recent decades, with an estimated lifetime prevalence ranging between 1.5% in Taiwan and approximately 18% in the United States (Krishnan 2003; Kessler and Bromet, 2013). Based on estimates from the World Health Organization, major depressive disorder accounts for the greatest proportion of burden associated with nonfatal health outcomes and contributes 34.1 million total years lived with disability (GBD 2017 Disease and Injury Incidence and Prevalence Collaborators, 2018). The definite pathophysiology of major depressive disorder is unclear, and it appears to have a complex etiology. Multiple genetic and environmental factors act together to develop a spectrum of neurobiological vulnerabilities to depression (Menard, Hodes et al., 2016).

The neurofilament light chain (NfL) is a subunit of neurofilaments that confers structural stability to neurons and is present in dendrites and the neuronal soma as well as in axons, where their expression is particularly high (Gaetani, Blennow et al., 2019). In response to central nervous system neuroaxonal damage caused by neuroinflammatory, neurodegenerative, traumatic, or vascular injuries, the release of NfL sharply increases, and NfL reaches the interstitial fluid, which communicates freely with the cerebrospinal fluid and the blood (Disanto, Barro et al., 2017; Gaetani, Blennow et al., 2019). Disanto et al. (2017) reported that NfL concentration in the blood is approximately 40-fold lower than that in the cerebrospinal fluid. NfL has been reported to serve as a molecular surrogate biomarker of neuroaxonal injuries in various neurological diseases, such as multiple sclerosis, Alzheimer’s disease, and traumatic brain injury (Zetterberg 2016; Disanto, Barro et al., 2017; Gaetani, Blennow et al., 2019). Mattioli et al. (2020) further demonstrated that higher levels of NfL were associated with greater cognitive impairment in patients with multiple sclerosis. Chatterjee et al. found an inverse association between NfL levels and cognitive function measured using a battery of neuropsychological tests in the healthy elderly population without dementia (Chatterjee, Goozee et al., 2018). Furthermore, regarding the association between NfL (as a biomarker of neuroaxonal injury) and proinflammatory cytokines (as a marker of neuroinflammation or systemic inflammation), Lu et al. reported a positive relationship between NfL levels and tumor necrosis factor (TNF)-α levels in patients with amyotrophic lateral sclerosis (Lu, Allen et al., 2016). Using the human brain aggregate cultures, Rempel et al. further demonstrated that interleukin (IL)-1β, a proinflammatory cytokine secreted by activated microglia, significantly increased NfL expression in primary neurons (Rempel, Kusdra et al., 2001). Evidence from the aforementioned studies may imply that neuroaxonal injury and neuroinflammation synergically resulted in the development of neurodegenerative disorders, such as amyotrophic lateral sclerosis, and the impairment of cognitive function (Rempel, Kusdra et al., 2001; Lu, Allen et al., 2016).

Increasing evidence suggests the presence of neuroinflammation and neuroaxonal damage in major depressive disorder (Csabai, Wiborg et al., 2018; Duarte-Silva, Macedo et al., 2019; Williams, Sharma et al., 2019). Williams et al. measured the density of myelinated axons between patients with major depressive disorder and healthy control participants and found a clear decrease in myelin in the axons of the callosal splenium in patients with major depressive disorder compared with controls (Williams, Sharma et al., 2019). An animal study of a chronic stress model for depression revealed that chronic stress reduced the number of synapses and myelinated axons in the infralimbic cortex (Jakobsson, Bjerke et al., 2014). However, studies on the association between NfL and major affective disorders are limited, with conflicting results (Jakobsson, Bjerke et al., 2014; Besse, Belz et al., 2020; Liu, Bavato et al., 2021). Jakobsson et al. found increased NfL levels in moderately ill patients with bipolar disorder (Jakobsson, Bjerke et al., 2014), but Besse et al. demonstrated that NfL concentrations did not differ between patients with major depressive disorder and healthy control participants (Besse, Belz et al., 2020). Liu et al. suggested that lifetime history of major depressive disorder was related to high NfL blood levels in patients with ketamine use disorder (Liu, Bavato et al., 2021). In addition, the detrimental impact of proinflammatory cytokines, such as TNF-α and IL-6, in major depressive disorder has been confirmed (Bai, Su et al., 2015; Bai, Chen et al., 2020; Chen et al., 2020b). Evidence indicated that executive deficits associated with frontal lobe dysfunction may be prominent in major depressive disorder (Fossati, Ergis et al., 2002). Executive function refers to cognitive processes that control and integrate other cognitive activities such as episodic memory (Fossati, Ergis et al., 2002). Our previous study suggested that elevated levels of TNF-α were related to poor executive function as measured using the Wisconsin Card Sorting Test (WCST) in patients with depressive disorder (Chen et al., 2020b). Ye et al. also revealed that increased IL-6 levels were implicated in the impairment of sustained attention in patients with major depressive disorder (Ye, Yin et al., 2018). However, the association between NfL, proinflammatory cytokines, and cognitive function in major depression remains unknown.

In the present study, following the evidence of neuroaxonal injury in major depressive disorder, NfL as a biomarker of neuroaxonal injury in various neurological diseases, and a positive association between NfL and proinflammatory cytokines, we examined the levels of NfL and proinflammatory cytokines, including TNF-α and IL-6, and assessed the cognitive function in patients with major depressive disorder. We investigated the association between NfL, proinflammatory cytokines, and cognitive function in patients with major depressive disorder, and we hypothesized that NfL is a novel biomarker for major depression and is associated with the cognitive dysfunction, such as executive dysfunction, resulting from major depression.

## METHODS

### Inclusion Criteria for Patients With Major Depressive Disorder and the Control Group

Patients aged between 13 and 64 years who met the Diagnostic and Statistical Manual of Mental Disorders, Fifth Edition criteria for major depressive disorder were enrolled as the study group. The enrolled patients had no major medical or neurological diseases or history of traumatic brain injury and alcohol or substance use disorders. Age- and sex-matched healthy controls who did not have a Diagnostic and Statistical Manual of Mental Disorders diagnosis, who were not pregnant or breastfeeding, and who did not have any major medical or neurological diseases (i.e., epilepsy, stroke, traumatic brain injury, or systemic autoimmune diseases) or unstable physical illnesses were recruited from the community and enrolled as the control group. A diagnosis of major depressive disorder was determined using the Mini International Neuropsychiatric Interview for adult patients, and the Kiddie Schedule for Affective Disorders and Schizophrenia for adolescent patients (Sheehan, Lecrubier et al., 1998; Chen, Shen et al., 2017). For all participants, demographic characteristics—including duration of illness, education, and body mass index (BMI)—were recorded, and clinical assessment, namely the 17-item Hamilton Depression Rating Scale (HDRS), was conducted. This study was approved by the Institutional Review Board of Taipei Veterans General Hospital and conducted in accordance with the Declaration of Helsinki. Written informed consent was obtained from all participants and their guardians prior to their inclusion in the study.

### Measurement of NfL

Plasma concentration of NfL was measured by quantitative horseradish peroxidase enzyme-linked immunosorbent assay (ELISA) kit (OKCD01380; Aviva Systems Biology, San Diego, CA). This assay was based on a standard sandwich ELISA method. An antibody specific for NfL was pre-coated on a 96-well plate, then 100 μL of standard or diluted samples (fourfold dilution) were added into different wells and incubated for 1 hour at 37°C. After removal of the standards and samples, a biotinylated detector antibody specific for NfL was added into each well and incubated at 37°C for 1 hour. Following 3 times of buffer washing, avidin-peroxidase conjugate was then added and incubated at 37°C for 30 minutes. The unbound conjugate was washed away using wash buffer for 5 times. An enzymatic reaction was produced through the addition of 3,3’,5,5’-tetramethylbenzidine substrate, which was catalyzed by horseradish peroxidase enzyme and generated a blue color product that changed to yellow after adding 50 μL of acidic stop solution. The optical density of yellow coloration was read at 450 nm of absorbance with a SpectraMax M2e microplate reader (Molecular Devices, Sunnyvale, CA) within 5 minutes and was quantitatively proportional to the amount of NfL captured in standards and sample wells. The NfL concentration was calculated based on a standard curve, which was linearized by plotting the log of the human NfL concentrations between 1.56 and 100 pg/mL vs the log of the optical density, and the best fit line was obtained from regression analysis.

### Measurement of Proinflammatory Cytokines

Proinflammatory cytokines, including IL-6, TNF-α, and CRP, were assayed using ELISA kits (R&D Systems, Minneapolis, MN) for all participants. Fasting serum samples were collected in serum separator tubes, clotted for 30 minutes, and stored at −80°C until use. All assays were performed according to the vendor’s instructions. The final absorbance of each sample of the mixture was measured and analyzed at 450 nm using an ELISA plate reader with Bio-Tek Power Wave Xs and Bio-Tek’s KC junior software (Winooski, VT). The standard range was considered as specified in the vendor’s instructions. A linear regression R-square value of at least 0.95 was considered a reliable standard curve.

### Assessment of Cognitive Function

In the current study, WCST was examined for executive function. WCST required strategic planning, organized searching, utilizing environmental feedback to shift cognitive sets, directing behavior towards achieving a goal, and modulating impulsive responding. The WCST task was commonly used in our previous studies (Chen, Li et al., 2018; Chen et al., 2020b; Hua, Chen et al., 2021).

### Statistical Analysis

The 1-way ANOVA for continuous variables and Fisher’s chi-square tests for nominal variables were used to assess differences between demographic and clinical data in the groups. Kolmogorov–Smirnov tests indicated that proinflammatory cytokines (IL-6, TNF-α, and CRP) were not normally distributed; they were then log-transformed. General linear models (GLMs) with the adjustment of demographic data (age, sex), groups, BMI, and clinical symptoms (HDRS) were performed to examine the association between NfL and proinflammatory cytokines (IL-6, TNF-α, and CRP) levels. Further GLMs with an additional adjustment of education and proinflammatory cytokines were used to assess the relationship between NfL levels and executive function measured by WCST. A 2-tailed *P* < .05 was considered statistically significant. All data processing and statistical analyses were performed using the SPSS version 17 software (SPSS Inc.).

## RESULTS

Overall, 40 patients with major depressive disorder and 40 age- and sex-matched controls were enrolled, and the sample had female predominance (Table 1). Patients with major depressive disorder had a lower education level (*P* = .039), higher scores of HDRS (*P < *.001), and higher levels of NfL (*P* = .007) than the controls (Table 1). The log-transformed levels of IL-6, TNF-α, and CRP did not differ between the 2 groups (Table 1). Finally, patients with major depressive disorder had greater cognitive deficits in WCST (all *P < *.05) compared with the controls (Table 1).

**Table 1.** Demographic Characteristics and Levels of NfL and Proinflammatory Cytokines Between Patients With Major Depressive Disorder and Controls

	Patients with major depressive disorder (n = 40)	Control group (n = 40)	P
Age (y, SD)	28.25 (14.35)	28.25 (14.08)	.977
Sex (n, %)			1.000
Male	13 (32.50)	13 (32.50)	
Female	27 (67.50)	27 (67.50)	
Education (y, SD)	12.00 (2.66)	13.60 (3.32)	.039
BMI (SD)	22.99 (4.50)	22.82 (3.71)	.858
HDRS total scores	22.55 (5.34)	0 (0.00)	<.001
Duration of illness (y, SD)	1.53 (4.72)		
First episode (n, %)	32 (80%)		
Repeated episode (n, %)	8 (20%)		
NfL levels (pg/mL, SD)	28.76 (22.53)	16.65 (8.07)	.007
Proinflammatory cytokines (SD)			
Log CRP	2.86 (0.59)	2.70 (0.57)	.235
Log IL-6	4.49 (0.15)	4.50 (0.10)	.901
Log TNF-α	2.94 (0.13)	2.93 (0.08)	.568
WCST (SD)			
Percent perseverative errors	13.66 (8.83)	9.63 (5.66)	.020
Percent conceptual level responses	63.09 (21.84)	75.95 (14.04)	.003
No. of categories completed	4.83 (2.06)	5.73 (0.78)	.013
Trials to complete first category	27.03 (33.23)	14.43 (7.66)	.023

Abbreviations: BMI, body mass index; CRP, C-reactive protein; HDRS, 17-item Hamilton Depression Rating Scale; IL, interleukin; NfL, neurofilament light chain; TNF, tumor necrosis factor; WCST, Wisconsin Card Sorting Task.

**Figure 1.**
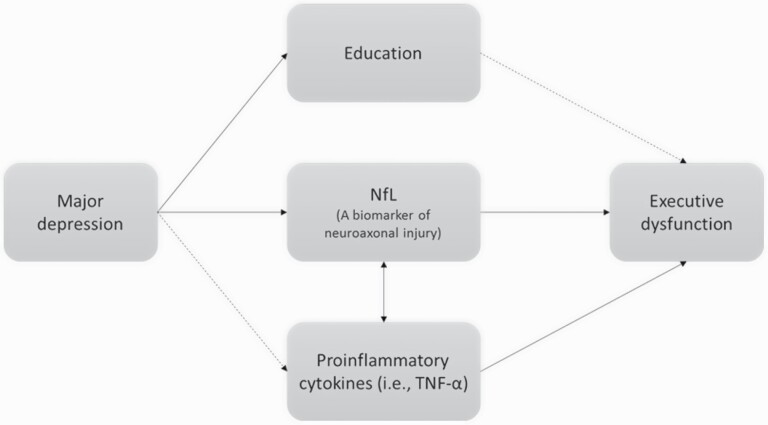
Study illustration of neurofilament light chain (NfL), proinflammatory cytokines, and cognitive dysfunction in depression (solid lines indicate the significance, and dashed lines indicate non-significance in current study). TNF, tumor necrosis factor.

GLMs with adjustment of demographic data, groups, BMI, and HDRS reported a significant association between plasma NfL concentrations and log-transformed levels of proinflammatory cytokines, particularly TNF-α (*P* = .004), but not CRP and IL-6 (Table 2). Finally, we examined the association between cognitive function, NfL, and proinflammatory cytokines and found that concentrations of NfL and TNF-α were associated with the deficits in executive function measured by WCST (Table 3). Figure 1 illustrated the association between NfL, proinflammatory cytokines, and cognitive dysfunction in major depressive disorder.

**Table 2.** GLMs for the Association Between NfL and Proinflammatory Cytokines^*a*^

	B	Std. Error	t	P
Intercept	−7.482	10.107	−0.740	.462
HDRS total scores	0.395	0.415	0.951	.345
BMI	0.242	0.445	0.545	.587
Log CRP	0.608	3.227	0.189	.851
Intercept	−107.630	53.648	−2.006	.049
HDRS total scores	0.492	0.406	1.212	.229
BMI	0.194	0.385	0.503	.617
Log IL-6	22.959	12.048	1.906	.061
Intercept	−134.227	44.143	-3.041	.003
HDRS total scores	0.557	0.394	1.415	.161
BMI	0.044	0.379	0.116	.908
Log TNF-α	45.670	15.495	2.947	.004

Abbreviations: BMI, body mass index; CRP, C-reactive protein; GLM, general linear model; HDRS, 17-item Hamilton Depression Rating Scale; IL, interleukin; TNF, tumor necrosis factor.

^*a*^Adjusted for age, sex, group, BMI, and HDRS total scores.

**Table 3.** GLMs for the Association Between NfL and Cognitive Function Measured by WCST ^*a*^

	B	Std. Error	t	P
WCST: percent perseverative errors				
Intercept	−42.181	22.927	−1.840	.070
Education	−0.494	0.261	−1.897	.062
Log TNF-α	15.932	8.058	1.977	.052
NfL	−0.004	0.060	-0.064	.949
WCST: percent conceptual level responses				
Intercept	216.760	53.997	4.014	<.001
Education	0.540	0.614	0.881	.382
Log TNF-α	−44.328	18.979	−2.336	.023
NfL	−0.198	0.142	−1.399	.166
WCST: no. of categories completed				
Intercept	17.903	4.702	3.807	<.001
Education	0.027	0.053	0.509	.612
Log TNF-α	−4.199	1.653	−2.541	.013
NfL	−0.025	0.012	−2.023	.047
WCST: trials to complete first category				
Intercept	−133.233	73.234	−1.819	.073
Education	−1.226	0.832	−1.473	.145
Log TNF-α	47.526	25.740	1.846	.069
NfL	0.615	0.192	3.200	.002

Abbreviations: BMI, body mass index; GLM, general linear model; HDRS, 17-item Hamilton Depression Rating Scale; NfL, neurofilament light chain; TNF, tumor necrosis factor; WCST, Wisconsin Card Sorting Task.

^*a*^Adjusted for age, sex, group, education, BMI, TNF-α levels and HDRS total scores.

## Discussion

Our findings supported the study hypothesis that patients with major depressive disorder exhibited significantly higher levels of NfL than the control participants. Higher levels of NfL were related to increased concentrations of TNF-α and were associated with the deficits in executive function measured by WCST. In addition, elevated TNF-α levels were related to executive dysfunction.

Studies have suggested the pathophysiological role of neuroaxonal injury in the development of major depressive disorder (Liu and Tang 2018; Guedes, Kenney et al., 2020). Guedes et al. found that elevated levels of NfL after mild traumatic brain injury were associated with the subsequent development of depression and posttraumatic stress disorder (Guedes, Kenney et al., 2020). Maller et al. examined the pathological patterns of neuroaxonal injuries between patients with traumatic brain injury and those with major depressive disorder and demonstrated that 2 conditions shared a common pathology of white matter disintegrity, namely reduced fractional anisotropy, in the frontotemporal tract, corpus callosum, and internal capsule (Maller, Thomson et al., 2010). A preliminary study of 236 patients with ischemic stroke revealed that baseline CRP and NfL levels independently predicted the development of poststroke depression, which may indirectly indicate a vicious cycle of neuroaxonal injury and neuroinflammation in the development of depression (Zhao, Mo et al., 2020). A brain autopsy study of 16 patients with major depressive disorder, 23 with schizophrenia, and 20 control participants revealed a decrease in myelin in the axons of the callosal splenium in patients with major depression, but not in those with schizophrenia and in controls (Williams, Sharma et al., 2019). In addition, several lines of evidence reported a significant association between increased NfL levels and deficits in cognitive function, particularly in patients with traumatic brain injury and those with neurodegenerative disorders (Kumar and Cook, 2002; Mahar, Alosco et al., 2017). Kumar et al. indicated that injury to the white matter from diverse biological sources (i.e., trauma or neurodegeneration) may compromise neural connectivity through associated axonal injury or impaired conductivity, which further damages cognitive function (Kumar and Cook, 2002). Our study reported that NfL may serve as a biomarker for major depressive disorder and further revealed a prominent association between NfL and depression-related executive dysfunction.

The second major finding of our study was the positive relationship between NfL levels and TNF-α levels. Evidence suggests the pathophysiological role of proinflammatory cytokines, especially TNF-α, in the development of major depressive disorder and neuroaxonal injury (Kita, Tanaka et al., 2000; Raison, Borisov et al., 2010; Kaster, Gadotti et al., 2012; Chio, Chang et al., 2013). An animal study on 6-week-old wild mice that were intracerebroventricularly given TNF-α revealed a depression-like behavior in the forced swimming test and tail suspension test (Kaster, Gadotti et al., 2012). Raison et al. reported that baseline TNF-α levels independently predicted depression onset in patients with hepatitis C who were treated with interferon-α (Raison, Borisov et al., 2010). Kita et al. investigated the potential injury of TNF-α in the axons in a midline fluid percussion rat model and demonstrated that TNF-α directly induced primary demyelination and oligodendrocyte apoptosis and contributed to the formation of delayed axonal damage (Kita, Tanaka et al., 2000). Chio et al. found that etanercept, a TNF-α antagonist, may attenuate traumatic axonal injury in rats by reducing early microglial expression of TNF-α (Chio, Chang et al., 2013). As mentioned, a positive relationship was observed between NfL levels and TNF-α levels in amyotrophic lateral sclerosis (Lu, Allen et al., 2016). Ouédraogo et al. further demonstrated that increased levels of CD4 T-cell–related proinflammatory cytokines (i.e., IL-17, TNF-α), but not anti-inflammatory cytokines (i.e., IL-10, IL-4), were related to NfL levels in patients with drug-resistant epilepsy (Ouedraogo, Rebillard et al., 2021). Our finding of a positive relationship between NfL levels and TNF-α levels may imply that neuroaxonal injury and neuroinflammation synergically contribute to the development of major depressive disorder.

Finally, our study reconfirmed the relationship between TNF-α and executive dysfunction in major depressive disorder (Peters, Ren et al., 2019; Chen et al., 2020a; Chen et al., 2020b). A study that assessed serum levels of IL-6 and TNF-α in 70 patients with major depressive disorder who completed the Behavior Rating Inventory of Executive Function found that higher TNF-α levels were associated with executive function deficits in inhibitory control (Peters, Ren et al., 2019). Bobińska et al. further suggested that elevated gene expression of TNF-α was correlated with cognitive deficits in working memory, executive function, attention, and auditory-verbal memory among patients with major depressive disorder (Bobinska, Galecka et al., 2017). However, surprisingly, we found no association between education and executive function, which may be related to the collinearity of major depressive disorder and education.

### Limitations

Several study limitations should be addressed. First, our study was a cross-sectional study and could not clarify the temporal association between NfL and proinflammatory cytokines, despite Rempel et al’s findings suggesting a significantly elevated NfL expression in primary neurons after treatment with IL-1β (Rempel, Kusdra et al., 2001). A prospective study is necessary to elucidate whether neuroaxonal injury (NfL) or neuroinflammation (i.e., TNF-α) occurs first. Second, only the WCST was employed for measuring cognitive function in the present study. Additional studies may be required to more comprehensively evaluate cognitive function in patients with major depressive disorder. Third, the medications used by the patients with major depressive disorder were not discontinued during the cognitive function assessment and the cytokine and NfL examination in the present study. In addition, previous studies suggested that antidepressants may exhibit the anti-inflammatory effect in patients with major depressive disorder (Hung, Huang et al., 2016, Galecki, Mossakowska-Wojcik et al., 2018), but no study ever investigated the potential role of antidepressants in the NfL. Allowing the patients to continue their medications was more ethical and prevented disease exacerbation; additionally, it enabled the collection of more naturalistic data. However, a drug-free study design may be required to confirm our findings. Fourth, owing to the difference of depression prevalence between Taiwan and United States and other countries, whether our results may be generalized to other ethnicities would need further investigation.

## CONCLUSION

In conclusion, NfL, which is related to neuroaxonal damage, may be a novel biomarker for major depressive disorder, which may correspond to the evidence of neuroaxonal injury in major depressive disorder. NfL concentrations were positively associated with the increased levels of proinflammatory cytokines, especially TNF-α. Increased levels of NfL and TNF-α were related to executive dysfunction in major depressive disorder.

## Interest Statement

All authors have no financial relationships relevant to this article to disclose.
